# Pathophysiological, Translational, and Diagnostic Aspects of ME/CFS: A Focus on Skeletal Muscle Involvement

**DOI:** 10.3390/diagnostics16071019

**Published:** 2026-03-28

**Authors:** Giorgio Fanò-Illic, Francesco Coscia, Paola V. Gigliotti, Franco Checcaglini, Ugo Carraro, Stefania Fulle, Rosa Mancinelli

**Affiliations:** 1 Department of Neuroscience, Imaging and Clinical Sciences, University “G. d’Annunzio” of Chieti-Pescara, 66100 Chieti, Italy; fanoillic@gmail.com (G.F.-I.); stefania.fulle@unich.it (S.F.); 2 IIM—Interuniversity Institute of Myology, University “G. d’Annunzio” of Chieti-Pescara, 66100 Chieti, Italy; 3 Campus of Free University of Alcatraz, Free University of Alcatraz, Santa Cristina di Gubbio, 06024 Gubbio, Italy; francesco.coscia1@gmail.com (F.C.); pao.gigliotti@gmail.com (P.V.G.); franco.checcaglini@libero.it (F.C.); 4 A&C M-C Foundation for Translational Myology, 35100 Padova, Italy; ugo.carraro@unipd.it; 5 Sports Medicine Service of the San Candido, Innichen and Brunico-Bruneck Hospitals, Bolzano-Bozen, 39038 San Candido, Italy; 6 Department of Biomedical Sciences, University of Padova, 35131 Padua, Italy

**Keywords:** oxidative stress, skeletal muscle, mitochondria, chronic fatigue, post-exertional malaise, Long COVID, E-C coupling dysregulation, redox imbalance, pacing

## Abstract

Myalgic Encephalomyelitis/Chronic Fatigue Syndrome (ME/CFS) is a chronic, multisystemic disorder characterized by severe, persistent fatigue not alleviated by rest and worsened by minimal exertion, often accompanied by post-exertional malaise (PEM), unrefreshing sleep, cognitive dysfunction, and autonomic disturbances. Despite decades of research, its pathophysiology remains incompletely understood, and skeletal muscle involvement has only recently gained attention. This review aims to provide a historical and pathophysiological synthesis of ME/CFS, emphasizing the pivotal role of skeletal muscle in the onset and persistence of symptoms, and to integrate molecular, cellular, and pathophysiological evidence into a coherent explanatory framework. This is a narrative review of published literature (1990–2025) with critical integration of clinical, biochemical, and experimental data on oxidative stress, mitochondrial dysfunction, Excitation–Contraction (E-C coupling) dysregulation, and muscle secretome alterations in ME/CFS also in relation to post-viral syndromes (e.g., Long COVID). Evidence consistently points to mitochondrial oxidative stress, redox imbalance, impaired Ca^2+^ handling, and altered signaling pathways in skeletal muscle of patients with ME/CFS. Historical milestones show an evolution from psychogenic interpretations toward recognition of ME/CFS as a biological disorder with neuromuscular and metabolic underpinnings. ME/CFS can be interpreted as a skeletal muscle–metabolic disorder characterized by oxidative distress, mitochondrial dysfunction, and impaired energy regulation, leading to the clinical picture of exercise intolerance and post-exertional malaise. Integrating basic and clinical research through a translational approach provides the foundation for new diagnostic tools, targeted therapies, and biomarkers.

## 1. Prologue

This review is not a conventional review but rather a synthesis of several decades of translational research carried out by our group and by other investigators, which progressively redefined ME/CFS as a biological, multisystemic disease. The focus is on skeletal muscle, long considered a “target organ,” but now increasingly viewed as an active player in the generation and persistence of the symptomatology.

The main aim of our research, initiated many years ago in the framework of the Interuniversity Institute of Myology (IIM) and later extended through collaborative studies with the A&C Mioni-Carraro Foundation for Translational Myology (A&C M-C Foundation for Translational Myology), has been to clarify the cellular and molecular mechanisms underlying muscle dysfunction in ME/CFS.

Over the years, our group contributed to demonstrating that muscle alterations in ME/CFS are not secondary to deconditioning but represent primary biochemical and structural abnormalities. In particular, we identified evidence of oxidative stress, mitochondrial dysfunction, and altered calcium homeostasis in skeletal muscle biopsies from affected patients [1,2,3].

These findings, later confirmed by other authors, have provided a biological rationale for the characteristic post-exertional malaise (PEM) and persistent muscle fatigue typical of the disease. The convergence of physiological, biochemical, and molecular evidence supports a unifying interpretation in which oxidative stress, mitochondrial impairment, energy supply deficit and E-C coupling dysregulation form a pathophysiological triad that links muscle dysfunction to systemic manifestations.

To prepare this narrative review, we conducted a comprehensive search on PubMed, Scopus, and Web of Science databases for articles published up to July 2025. Search terms included “ME/CFS”, “skeletal muscle”, “mitochondrial dysfunction”, “oxidative stress”, Long-COVID and “myokines”. We included peer-reviewed original studies and reviews focusing on clinical and molecular findings in patients meeting established diagnostic criteria (e.g., CCC, IOM). Case reports and studies with non-specific “chronic fatigue” definitions were excluded.

## 2. The Definition and Management of Myalgic Encephalomyelitis

**Figure 1:**
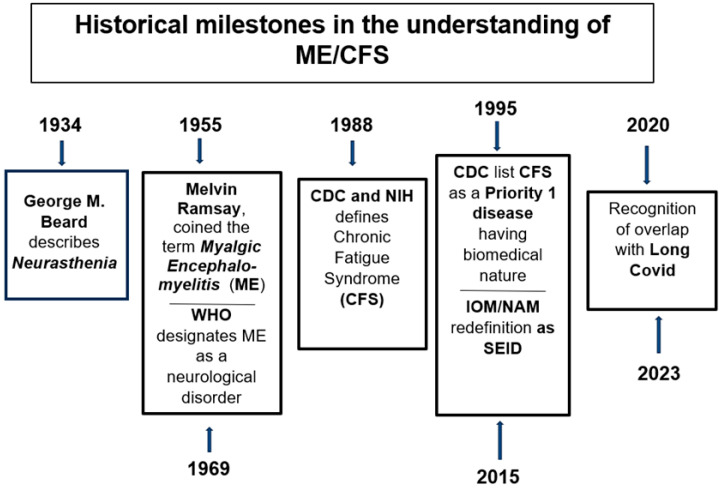
Historical milestones in the understanding of ME/CFS. The timeline summarizes major scientific and clinical milestones in the recognition of ME: from Beard’s description of Neurasthenia (1880) and the Los Angeles outbreak (1934), through to the Royal Free Hospital epidemic (1955) and WHO neurological classification (1969), the CDC case definitions (1988 and 1995), the IOM/NAM redefinition as SEID (2015), and the recent overlap with Long COVID (2020–2023).

The history of ME/CFS is long, complex, and often controversial (Figure 1). It spans more than a century of debate, misunderstanding, and gradual scientific recognition. In Western countries—the most affected regions globally—at least five million individuals are diagnosed with ME/CFS (more than twelve million worldwide), predominantly women in middle adulthood [4]. The first description of a clinical condition resembling ME/CFS dates back to the late nineteenth century, when the American neurologist and neuropsychiatrist George Miller Beard introduced the term neurasthenia to define a state of easy fatigability and nervous exhaustion accompanied by numerous nonspecific symptoms—ranging from insomnia to mood changes and impaired concentration—occurring without significant objective findings [5]. This clinical situation was most likely caused by what were clearly psychological reactions among nurses exposed to children with negative outcomes due to polio. A major event contributing to the conceptual evolution of the disorder occurred in 1934, when an outbreak among healthcare personnel at the Los Angeles County Hospital—initially confused with poliomyelitis—affected around 200 individuals. The syndrome was characterized by flu-like symptoms followed by profound muscle weakness, fatigue, and neurological signs. Although the illness was non-lethal, recovery was often prolonged, with persistent disability in many patients [6]. Another key episode took place in 1955 at the Royal Free Hospital in London, where more than 300 staff members, mostly women, developed a similar condition. The outbreak was meticulously described by Melvin Ramsay, who later coined the term myalgic encephalomyelitis to emphasize the neurological and inflammatory aspects of the disease [7]. In 1969, myalgic encephalomyelitis was officially included in the World Health Organization’s International Classification of Diseases (ICD) under neurological disorders [8]. In 1984, a new epidemic occurred in Incline Village, Nevada, originally attributed to chronic Epstein–Barr virus infection. The subsequent investigations by the Centers for Disease Control and Prevention (CDC) and the National Institutes of Health (NIH) led to the introduction of the term Chronic Fatigue Syndrome (CFS) (CDC, 1988) [9]. Skepticism and stigma followed, with some early commentaries dismissing it as “Yuppie Flu” or “a disease of depressed menopausal women” [10]. Only in 1995 did the CDC list CFS as a Priority 1 disease among “new and re-emerging infectious diseases,” formally acknowledging its biomedical nature. The 1994 CDC case definition required:

1. Persistent or relapsing fatigue lasting at least six months, not alleviated by rest, resulting in substantial reduction in activity;
2. The presence of at least four out of eight associated symptoms, e.g., impaired memory or concentration, sore throat, tender lymph nodes, muscle pain, multi-joint pain, unrefreshing sleep, and post-exertional malaise (PEM).

In 2015, the Institute of Medicine (now the National Academy of Medicine) proposed revised diagnostic criteria under the label Systemic Exertion Intolerance Disease (SEID), requiring substantial activity reduction, post-exertional malaise, and unrefreshing sleep, plus either cognitive impairment or orthostatic intolerance [11]. Today, the consensus within the scientific community increasingly recognizes ME/CFS as a biological, multisystemic disorder involving complex interactions among the immune, nervous, and metabolic systems [12,13]. In addition, the emergence of Long COVID, with its overlapping symptoms and mechanisms, has further revitalized interest in ME/CFS pathophysiology [14,15]. Currently, the complex situation regarding the criteria used to define pathological status (Fukuda/CDC 1994, Canadian Consensus Criteria/CCC, 2003 and IOM/SEID, 2015) can be summarized as shown in Table 1.

The distinctive features of the three methodologies listed in the table can be summarized as follows:

1. Fukuda Criteria (1994 CDC).

These criteria require at least 6 months of persistent fatigue accompanied by 4 out of 8 specific minor symptoms. They do not mandate the obligatory presence of Post-Exertional Malaise (PEM), which is now recognized as the hallmark of the disease. Consequently, the specificity is low: a significant number of patients with mood disorders or other idiopathic fatigue syndromes may be misdiagnosed with ME/CFS under this framework.

2. Canadian Consensus Criteria (CCC 2003)

The CCC requires the presence of PEM, pain, sleep disturbances, and at least two neurological/cognitive symptoms, in addition to symptoms from at least two categories among autonomic, neuroendocrine, or immune manifestations. The greatest strength of this procedure is represented by high specificity. These criteria identify a subset of patients with significantly higher physical disability and more pronounced biological abnormalities (biomarkers).

3. IOM Criteria (IOM/SEID, 2015)

Proposed under the name SEID, this framework requires three core symptoms (fatigue, PEM, and unrefreshing sleep) plus either cognitive impairment or orthostatic intolerance. This procedure is well balanced and validation studies indicate that IOM criteria maintain high sensitivity while substantially improving specificity by excluding false positives primarily linked to psychiatric comorbidities.

However, it is necessary to note that while the IOM criteria are currently favored in clinical practice due to their streamlined application, many researchers continue to utilize the **CCC** for selecting homogeneous study cohorts. This ensures that participants exhibit the multisystemic dysfunctions characteristic of Myalgic Encephalomyelitis [16]. In medical literature and international guidelines, PEM is the hallmark symptom of ME/CFS. The National Academy of Medicine (NAM/IOM) defines it as “An exacerbation of some or all of an individual’s ME/CFS symptoms after exposure to physical or cognitive stressors that were normally tolerated before disease onset.” while by NICE Guidelines [17]. PEM is “The worsening of symptoms which is often delayed in onset by hours or days, is disproportionate to the activity, and has a prolonged recovery time.”. For clinical trials, PEM is increasingly measured using the DePaul Symptom Questionnaire (DSQ-PEM) to ensure diagnostic rigor [18]. In this regard, attention has been drawn to a new questionnaire that has been proposed for the assessment of PEM. In clinical practice and in the literature, it is referred to as FUNCAP [19]. Two versions of the questionnaire were developed: a longer version comprising 55 questions (FUNCAP55), developed for improved diagnostic and disability benefit/insurance Functional Capacity assessments; and a shorter version (FUNCAP27) for clinical patient follow-up and potential use in research.

**Figure 2:**
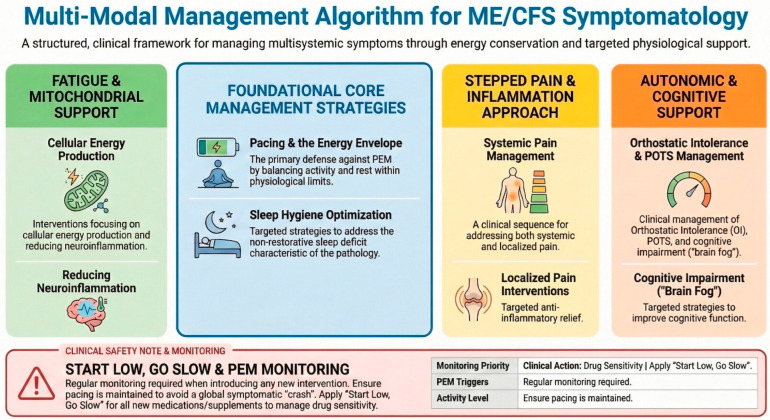
Multi-Modal Management Algorithm for ME/CFS Symptomatology.

The factors that trigger PEM onset can derive from physical, cognitive, emotional, or orthostatic (standing) stress. The onset can be immediate, but is characteristically delayed by 12–48 h. It does not improve with rest; typically takes >24 h to return to baseline. The consequence of this is a global “crash” involving neurocognitive, immune, and pain symptoms [20]. All clinical interventions must be applied according to the “Start Low, Go Slow” principle due to the high frequency of drug sensitivities in this patient population [11]. Regular monitoring for PEM triggers is essential when introducing any new supplement or medication. Due to the lack of specific pharmacological interventions, non-pharmacological strategies focusing on energy conservation are paramount. The cornerstone of PEM prevention is the “Pacing” a self-management strategy that involves carefully balancing activity and rest to stay within an individual’s unique physiological limits and avoid triggering PEM. It is not about increasing activity over time (as in graded exercise therapy, which is contraindicated in ME/CFS due to the risk of exacerbating PEM), but rather about consistent energy expenditure management [21]. Keeping these considerations in mind, it is possible to construct a flow chart that can be used as a model for the management and treatment of patients diagnosed with ME/CFS. Figure 2 shows the reference framework for a treatment that takes into account the muscular hypothesis of the symptoms expressed during the clinical course of ME/CFS.

- Core Management Strategies (Center Blue Box): Represents the foundational, non-pharmacological interventions required for all patients. Pacing and maintaining the Energy Envelope are the primary defenses against PEM. Sleep hygiene optimization targets the restorative sleep deficit characteristic of the pathology.
- Fatigue & Mitochondrial Support (Left Green Column): Focuses on cellular energy production and neuroinflammation.
- Pain & Inflammation (Center Yellow Column): Outlines a stepped approach to systemic and localized pain.
- Autonomic & Cognitive Support (Right Orange Column): Addresses Orthostatic Intolerance (OI) and POTS (Postural Orthostatic Tachycardia Syndrome), which are highly comorbid with ME/CFS.

All clinical interventions must be applied according to the “Start Low, Go Slow” principle due to the high frequency of drug sensitivities in this patient population. Regular monitoring for PEM triggers is essential when introducing any new supplement or medication [22].

## 3. Part One—Specific Muscle Damage

**Figure 3:**
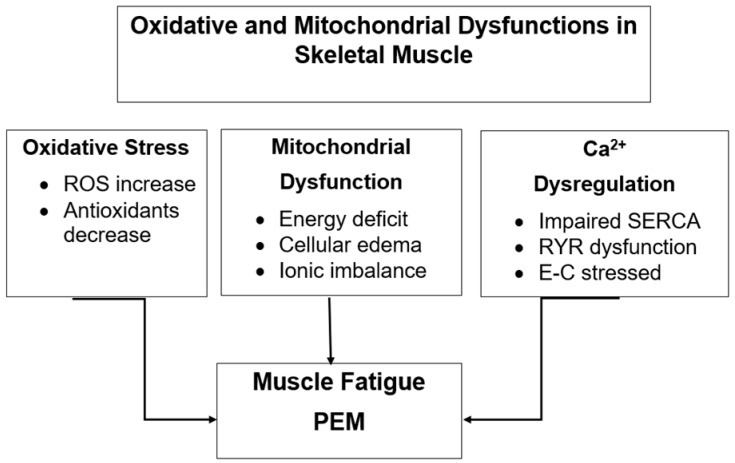
Oxidative and mitochondrial dysfunctions in skeletal muscle of patients with ME/CFS. The diagram illustrates the interrelated mechanisms of oxidative stress, mitochondrial dysfunction and calcium dysregulation observed in skeletal muscle of patients with ME/CFS. Increased ROS and decreased antioxidants induce mitochondrial damage and calcium handling abnormalities like Sarcoplasmic Reticulum Calcium Pump (SERCA), Ryanodine receptor (RyR), leading to impaired energy production, altered excitation–contraction cycles and the clinical manifestation of muscle fatigue and post-exertional malaise (PEM).

Since the early 1990s, the number of scientific papers addressing ME/CFS has increased considerably. Analysis of PubMed data shows that between 1990 and 2000, approximately 200 papers per year were published on the topic. However, the majority of these focused on the etiology and pathogenesis of the syndrome or on therapeutic approaches to its management. Only a small subset investigated biomolecular or tissue-level aspects, and none provided direct evidence of structural or functional abnormalities in skeletal muscle obtained from patients clinically diagnosed with ME/CFS. Two main reasons account for this gap. First, despite the efforts made by the scientific and clinical community to unequivocally characterize the presence of ME/CFS, the diagnosis of this condition remains too often linked to the concept of exclusion and assigned only after ruling out other medical conditions capable of causing chronic fatigue. Second, there were practical and ethical limitations in obtaining muscle biopsies from patients, as conventional sampling required surgical incisions and suction needles to retrieve tissue fragments. Our research team, in collaboration with the Neurology Service at Massachusetts General Hospital and Harvard Medical School (Boston, USA), was actively engaged in studies on age-related oxidative damage in skeletal muscle. Their research demonstrated that aging exhibits a progressive oxidative modification of macromolecules (proteins, lipids, DNA) due to a gradual decline in mitochondrial respiratory chain efficiency, leading to the accumulation of reactive oxygen species (ROS) (Figure 3). This damage can exceed the compensatory capacity of antioxidant defense systems [23,24]. The same group proposed that sarcopenia, a partially reversible age-related process, may result from an imbalance between oxidant and antioxidant systems, causing persistent oxidative stress and impaired calcium transport within muscle sarcoplasmic reticulum sites [25,26]. Additionally, it is important to note that also the reparative and regenerative capacities of adult stem cells derived from aged skeletal muscle were significantly altered if harvested from sarcopenic muscles [27,28]. A critical resource for this research was our muscle biobank which preserved muscle samples in liquid nitrogen from a variety of clinical studies. Among the archived material were biopsies from patients diagnosed with ME/CFS, collected and stored under standardized conditions. Analyses of these samples revealed, for the first time, evidence of specific oxidative damage in skeletal muscle of patients with ME/CFS—damage that was distinct from both healthy controls and from patients with fibromyalgia, another condition associated with chronic pain and fatigue [1]. Using subcellular fractionation and biochemical assays, this group demonstrated that the oxidative imbalance in ME/CFS muscle had direct functional consequences on key muscle structures, including the sarcoplasmic reticulum and mitochondrial compartments [29]. At that time, in vitro physiological testing of intact muscle fibers was impossible because most available biopsies had been obtained using the traditional needle suction technique, which fragmented muscle fibers and disrupted their structural integrity. To overcome this limitation, the group developed a novel, minimally invasive sampling method that allowed the extraction of intact muscle fragments without incisions or suction—a technique they termed Tiny Percutaneous Needle Biopsy (TPNB) [30]. This approach produced small, structurally preserved tissue samples, suitable for mechanical, morphological, and biochemical studies. The TPNB method was later validated through collaborative ultrastructural analysis with Clara Franzini-Armstrong at the University of Pennsylvania, confirming excellent preservation of sarcomeric and mitochondrial organization [31]. Compared to conventional surgical biopsies, TPNB offers several advantages—it requires no incision, leaves no visible scars, causes minimal discomfort, and enables repeated sampling over time—making it particularly valuable for longitudinal studies in ME/CFS patients.

By the early 2000s, our group findings on oxidative and functional alterations in ME/CFS muscle aligned with a growing number of studies reporting systemic oxidative stress markers in patient blood or serum:

- Elevated protein carbonyls, indicative of oxidative modification of proteins [31];
- Altered heat-shock protein responses to physical exertion [32];
- Increased lipid peroxidation [33].

Although limited in number, these studies converged on a working hypothesis: oxidative stress-induced molecular alterations in skeletal muscle may directly contribute to the persistent, non-restorative fatigue characteristic of ME/CFS [2]. More recently, investigations at Stanford University School of Medicine (USA) confirmed the role of oxidative stress as a unifying feature of ME/CFS and Long COVID, based on lymphocyte analyses from 20 Long COVID patients, 27 ME/CFS patients, and 25 controls. Both patient groups exhibited signs of oxidative imbalance, though with distinct molecular signatures [34]. The authors proposed molecular biomarkers for ME/CFS diagnosis and highlighted shared redox dysregulation between ME/CFS and post-viral fatigue syndromes. The relationship between oxidative stress and ME/CFS is therefore well-established, although the direction of causality remains debated. As proposed by Pietrangelo et al. [35] and later reaffirmed [36], skeletal muscle in ME/CFS behaves as though it were “an old muscle within a young body.” Other studies supported this concept by demonstrating mitochondrial RNA dysregulation and altered β-oxidation pathways in ME/CFS muscle [37,38]. More recently, quadriceps femoris biopsies from ME/CFS patients showed compromised oxidative phosphorylation and progressive mitochondrial morphological abnormalities [39]. Earlier electron microscopy investigations [40] found no major structural mitochondrial defects but functional anomalies, an observation now corroborated by molecular evidence. A recent review by Scheibenbogen and Wirth (2025) [41] suggests that oxidative stress-induced mitochondrial dysfunction leads to impaired calcium homeostasis, particularly within subsarcolemmal mitochondria, resulting in elevated intracellular sodium and reduced handgrip strength (HGS)—markers of systemic fatigue [42]. The underlying mechanism may involve ROS-dependent Na^+^/K^+^-ATPase inhibition and sodium–calcium dysregulation within muscle fibers. Complementary transcriptomic analyses revealed differential expression of genes related to mitochondrial bioenergetics, oxidative balance, energy metabolism, and muscle trophism, including reduced transcription of the gene encoding the nicotinic acetylcholine receptor binding site [43]. Further studies of myosin heavy-chain (MHC) isoforms in vastus lateralis biopsies demonstrated a shift toward fast-fatiguing type 2X fibers, characterized by high ATP consumption and low endurance, while single-fiber contractile capacity remained preserved [44]. From a physiological standpoint, these alterations fit within the conceptual framework of allostasis, whereby multiple cellular sensors dynamically monitor and respond to environmental and functional challenges. When adaptation succeeds, a state of eustress emerges; when it fails, distress arises [45]. Mitochondria serve as central regulators of this adaptive signaling network [46]. Our group expanded this model by proposing that the H_2_O_2_/Ca^2+^/Zn^2+^ complex acts as a collaborative redox sensor coordinating mitochondrial capacity [47]. Histological analyses found no evidence of ischemic myopathy, immune-mediated inflammation, or viral inclusion, suggesting that mitochondrial and ionic alterations are primary functional disturbances rather than secondary consequences [41]. Taken together, these findings indicate that skeletal muscle in ME/CFS is trapped in a self-perpetuating loop of oxidative and mitochondrial dysfunction, leading to diminished energy output—an explanation consistent with exercise intolerance and post-exertional malaise (PEM) [48,49]. Finally, handgrip strength (HGS) studies show a strong inverse correlation between muscle fatigue and overall disease severity [42], suggesting that mitochondrial impairment extends across multiple muscle groups rather than being localized to the quadriceps.

## 4. Part Two—Can the Symptomatology of ME/CFS Patients Originate from Muscle?

The hypothesis that skeletal muscle plays an active and possibly primary role in the pathogenesis and symptomatology of ME/CFS has gained increasing support in recent years. Skeletal muscle should no longer be viewed merely as a mechanical effector of movement, but rather as a complex endocrine and autocrine/paracrine organ capable of releasing a wide array of signaling molecules that influence distant tissues and organs [50,51].

### 4.1. The Muscle as a Secretory Organ

Research in the past two decades has led to a paradigm shift: skeletal muscle is now recognized as a secretory organ that communicates bidirectionally with the immune, nervous, endocrine, and other metabolic systems through the release of myokines, extracellular vesicles (EVs), and microRNAs (miRNAs) [52,53,54]. This network of secreted factors, collectively termed the muscle secretome, plays a fundamental role in systemic homeostasis, modulating inflammation, glucose metabolism, angiogenesis, neuroplasticity, and even bone remodeling [55]. Among the earliest identified myokines, interleukin-6 (IL-6) occupies a prominent position. Initially considered a pro-inflammatory cytokine, IL-6 is now recognized as a dual-function molecule: when released transiently during muscle contraction, it exerts anti-inflammatory and metabolic effects, enhancing glucose uptake and lipid oxidation; under chronic conditions, however, it contributes to systemic low-grade inflammation [56,57,58]. Further studies identified additional myokines—such as irisin, myostatin, decorin, SPARC, brain-derived neurotrophic factor (BDNF), fibroblast growth factor 21 (FGF21), and CXCL1—each with distinct roles in regulating metabolism, cell differentiation, and tissue repair and also in muscle-to-tumor crosstalk [59,60,61].

### 4.2. Extracellular Vesicles and miRNAs in Muscle Communication

EVs, nanosized lipid bilayer-bound particles, carry proteins, lipids, and nucleic acids, including miRNAs. These vesicles mediate inter-tissue communication, affecting immune responses, vascular integrity, and neuromuscular signaling [54]. In ME/CFS, proteomic studies of circulating EVs have revealed substantial alterations in their molecular cargo, particularly following exertion. In a landmark study, Giloteaux et al. (2024) [62] compared serum EVs before and after maximal exercise in women with ME/CFS and healthy controls, proteomic analysis indicated that several modified proteins were linked to muscle contraction and others are related to the immune system (inflammation?) or brain signalling (cognitive aspects?). In addition, the changes observed were correlated with the severity of the disease [62]. Results consistent with those obtained in female patients were obtained from Glass et al. (2025) [63] in male patients compared with control subjects. Again, after exercise, proteomic analysis of EVs derived from the plasma of patients with ME/CFS showed alterations in the protein pattern involved in the immune system and in the system controlling certain brain functions, such as the sleep/wake cycle. In males, as was evident in females, protein alterations were proportional to the severity of the pathological condition [63]. These findings strongly suggest that exercise-induced EV alteration contributes to post-exertional malaise (PEM) and to the persistence of systemic symptoms by perpetuating inflammatory and metabolic dysregulation [64].

### 4.3. Muscle Secretome Dysregulation and Systemic Manifestations

Muscle-derived secretory molecules also include S100 proteins involved in calcium signaling, neurotrophins such as BDNF and NGF, and regulatory peptides like myostatin, which inhibits myogenesis, and decorin, which promote muscle regeneration. Exercise normally modulates their expression dynamically, balancing anabolic and catabolic signals [65]. In ME/CFS, however, this regulation could be profoundly altered but it is difficult to understand whether this is directly related to the pathology or caused by the reduction in physical activity imposed by pacing, which is normally used as elite therapy in ME/CFS patients. One thing is certain: in patients suffering from Long COVID who show symptoms very similar to ME/CFS, myokine secretion is significantly altered [66]. Proteomic data further indicate that exercise in male patients with ME/CFS—instead of activating adaptive myokine signaling—leads to maladaptive EV release that exacerbates systemic inflammation and muscle metabolic imbalance [63]. To evaluated if muscle metabolic abnormalities might also occur during a programme of repeat exercise and using magnetic resonance spectroscopy (MRS) as methodological approach, Jones et al. demonstrate an excessive lactic acid accumulation in the muscle and slowed phosphocreatine recovery after exercise [67].

This may explain why even minimal exertion can worsen symptoms and prolong recovery in affected individuals.

### 4.4. Immune Dysregulation and the Role of Inflammation

A growing body of evidence, even if not all, demonstrates that immune dysfunction and chronic low-grade inflammation play crucial roles in the perpetuation of ME/CFS. The seminal study by Brenu EW et al. (2011) first described altered cytokine profiles in ME/CFS, with increased production of pro-inflammatory cytokines (IL-1β, IL-6, TNF-α) and reduced levels of anti-inflammatory cytokines (IL-10) [68]. Today, the role of immune system impairment and inflammatory mechanisms are pivotal to the initiation and advancement of ME/CFS [69]. It is also possible that oxidative stress may act as a molecular bridge between these immune abnormalities and muscle dysfunction. ROS-mediated damage in skeletal muscle triggers the release of damage-associated molecular patterns (DAMPs), which in turn activate Toll-like receptor (TLR) and inflammasome pathways, further amplifying immune activation and fatigue [70].

Cerebral inflammation is hypothesized to be involved in the pathophysiology of CFS/ME. Almutairi et al. conducted a systematic review of published studies using imaging techniques as MRI in ME/CFS patients, but did not reach any definitive conclusions [71]. Very recently, the development of new sensors used in near-infrared spectroscopy (NIRS), a technique that allows comparison with data obtained with the aid of NMR, leaves the door open to results that are more useful for a definite diagnosis, at least of the state of fatigue typical of the disease [72].

Another useful example of video imaging techniques being used to study stratification—which is necessary for defining the severity of ME/CFS—concerns the hypothesis that neuroinflammation is present also in the CNS specific area, and it is causally linked to the severity experienced by ME/CFS patients [73].

This possibility was directly checked using (11)C-(R)-PK11195 and PET [74]. Data demonstrated that the binding potential (BP(ND)) was 45% to 200% higher in the cingulate cortex, hippocampus, amygdala, thalamus, midbrain and pons of CFS/ME patients than in healthy controls but the cause/effect relation has not been proven in this case either.

### 4.5. Muscle as Both Target and Source of Symptoms

Taken together, many findings available support the view that skeletal muscle in ME/CFS is not merely a passive victim of systemic dysfunction but also an active participant in symptom generation. The altered muscle secretome, shaped by mitochondrial dysfunction and oxidative stress, likely contributes to the systemic manifestations of ME/CFS—such as fatigue, cognitive impairment, and dysautonomia—via disrupted communication between muscle, brain, and immune systems [75]. In addition, miRNA analyses of muscle biopsies from ME/CFS patients have shown differential expression of miRNAs regulating mitochondrial biogenesis, calcium homeostasis, and oxidative stress responses [76,77]. Interestingly, several of these miRNAs are also dysregulated in Long COVID, suggesting convergent post-viral fatigue mechanisms [78,79]. All these data further strengthen this interpretation: ME/CFS muscle fibers exhibit redox-dependent alterations in the sarcoplasmic reticulum and mitochondria, consistent with disturbed Ca^2+^ handling, ROS accumulation, and reduced contractile recovery. These cellular phenomena mirror the clinical triad of fatigue, post-exertional malaise, and delayed recovery, which defines ME/CFS (Table 2).

In ME/CFS, skeletal muscle acts as an active endocrine organ, but its secretome is dysregulated due to mitochondrial dysfunction. These signaling molecules (myokines, metabolites, ROS, Growth Factors and EVs) act as mediators of the “systemic crash,” providing a bridge between physical exertion and multi-systemic symptoms. These pathways also contribute to systemic inflammation, neurocognitive impairment, and metabolic dysregulation, which together underlie PEM and the multisystemic nature of ME/CFS. The post-exertional elevation of pro-inflammatory myokines and the aberrant cargo of extracellular vesicles provide a plausible biological mechanism for the systemic nature of PEM. Specifically, the enrichment of EVs with mitochondrial DNA (mtDNA) and specific miRNAs suggests a “danger signal” propagation mechanism: these vesicles may cross the blood–brain barrier, triggering microglial activation and subsequent neuroinflammation. This explains why peripheral muscle exertion leads to central symptoms such as cognitive impairment and “brain fog” [80]. Furthermore, the impaired clearance of lactate and the excessive production of ROS underscore a state of metabolic inflexibility. The activation of acid-sensing ion channels (ASICs) due to localized acidosis contributes to the chronic pain phenotype, while the reduced bioavailability of neurotrophic growth factors like BDNF and IGF-1 indicates a failure in the homeostatic recovery phase. Taken together, these findings suggest that targeting the skeletal muscle secretome—either through pharmacological stabilization or precision pacing—could represent a novel diagnostic and therapeutic frontier for the disease [81].

In conclusion, we can hypothesize that the skeletal muscle is both a target and a driver of the pathophysiological processes underlying ME/CFS. Its dual role—as an effector organ suffering oxidative and mitochondrial dysfunction, and as a secretory organ perpetuating systemic dysregulation—offers a coherent explanation for the chronicity, variability, and multisystemic nature of the disease.

## 5. Part Three—Final Considerations and Future Perspectives

The overall picture that emerges from this integrated analysis is that Myalgic Encephalomyelitis/Chronic Fatigue Syndrome can be conceptualized as a neuromuscular–metabolic disease, in which skeletal muscle plays a central role both as a target and as an active driver of systemic dysfunction. A growing body of molecular, histological, and functional evidence indicates that oxidative stress, mitochondrial dysfunction, and calcium mishandling converge to impair energy metabolism and redox homeostasis in muscle fibers. This combination of processes leads to a condition of energetic insufficiency that mirrors the physiological characteristics of premature muscle aging—the so-called “old muscle in a young body” hypothesis [35].

### 5.1. Oxidative Stress and Mitochondrial Dysfunction as Central Hubs

Mitochondria are at the heart of this pathological network. They represent the main source and target of reactive oxygen species and are critically involved in regulating ATP production, calcium buffering, and apoptotic signaling. Under physiological conditions, mitochondria maintain a fine balance between energy demand and redox signaling. In ME/CFS, however, chronic oxidative stress disrupts this equilibrium, resulting in decreased oxidative phosphorylation, excess ROS generation, and aberrant calcium cycling [41]. This dysfunctional interplay is further aggravated by alterations in the Na^+^/K^+^-ATPase pump and impaired sarcoplasmic reticulum Ca^2+^ reuptake, which together reduce muscle contractile efficiency and delay recovery after exertion [29,44,46].

### 5.2. A Vicious Cycle of Energetic Collapse

The sequence of biochemical events triggered by oxidative distress can be viewed as a vicious cycle:

1. Mitochondrial inefficiency increases ROS production.
2. ROS in turn impair calcium handling and ion transport.
3. This leads to further mitochondrial damage and energetic decline.

As we have highlighted in previous chapters, the “mitochondrial hypothesis” in ME/CFS suggests that the hallmark fatigue symptoms are results of bioenergetic failure. Research indicates that patients’ cells fail to switch efficiently from glycolysis to oxidative phosphorylation.

This self-perpetuating loop results in a sustained state of metabolic “lockdown” that prevents the muscle from restoring homeostasis even after minimal exertion. Clinically, this mechanism offers a coherent explanation for the PEM characteristic of ME/CFS [82].

### 5.3. Long COVID as a Model for Post-Viral ME/CFS

The advent of Long COVID has provided an unprecedented opportunity to study post-viral fatigue syndromes in real time. Numerous clinical and molecular parallels between Long COVID and ME/CFS have been documented, including mitochondrial dysfunction, oxidative imbalance, immune dysregulation, and autonomic instability [83,84,85]. However, not all Long COVID cases evolve into ME/CFS [86,87]. A key differentiating factor appears to be the pre-existing muscle conditioning of the individual: physically active patients show better recovery trajectories and less severe fatigue, likely due to enhanced mitochondrial resilience. Some studies on therapeutic attempts based on graded exercise therapy (GET) [88] or cognitive behavioral therapy (CBT) [89] are declared to be effective and safe for ME/CFS; others have proven largely ineffective—and in some cases detrimental—for these patients, as they may exacerbate oxidative stress and symptom severity [90,91]. A comparison carried out using MRI imaging confirmed that in long-COVID situations, the symptoms related to fatigue in both pathological states have similar findings [92].

### 5.4. Toward a Muscle-Centered Diagnostic and Therapeutic Model

**Figure 4:**
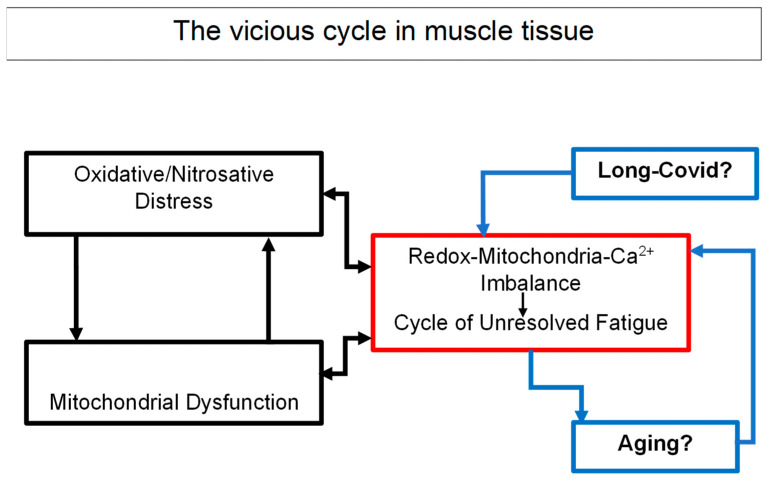
Schematic representation of ME/CFS pathophysiology in skeletal muscle. The diagram depicts the vicious cycle involving oxidative stress, mitochondrial dysfunction, and calcium imbalance, in which two or more elements intensify and aggravate each other, leading inexorably to skeletal muscle alterations to immune dysregulation, unresolved fatigue and the possible overlap with Long COVID and aging effects on muscle tissue.

The cumulative evidence supports a muscle-centered pathophysiological model of ME/CFS, in which skeletal muscle acts as a sentinel organ linking metabolic, immune, and neurological dysfunction. In this model, muscle-derived signals—mediated by ROS, Ca^2+^, Zn^2+^, and secreted myokines—drive systemic dysregulation, contributing to neuroinflammation, cognitive deficits, and autonomic symptoms (Figure 4).

Such a model provides a foundation for the development of novel diagnostic biomarkers, including:

- Blood-based measures of redox imbalance;
- Alterations in specific muscle-related microRNAs;
- EV-associated protein signatures reflecting mitochondrial distress.

**Figure 5:**
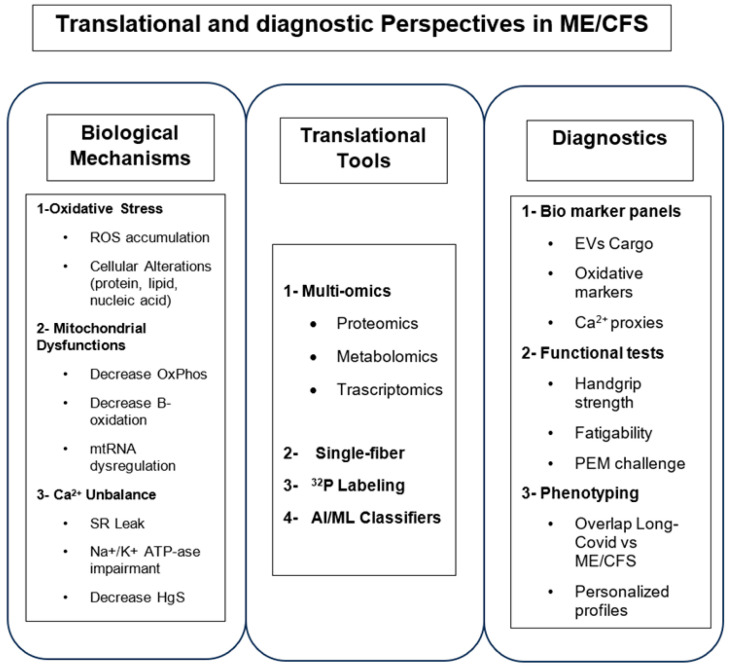
Schematic representation of translational and diagnostic perspectives in ME/CFS.

However, it is worth remembering that unfortunately, efforts to define a therapeutic approach for this condition have so far been disappointing, with no clear solution yet identified. The attempts including direct action on the vicious circle of altered energy production, which leads to the establishment of a state of oxidative stress that can further alter energy production, in large-scale trials has often been modest. Examples of this include the use of CoQ10, NADH, or antioxidants [93]. In recent years, researchers have tried to associate the muscular and/or neuromuscular symptoms of ME/CFS patients with those of apparently similar Long-COVID patients, despite the solid rational basis, but this strategy has yielded disappointing results, as any benefits are often limited to specific subgroups of patients. Very recently, Eckey M et al. analysed a very large group of ME/CFS patients in relation to subjects with equivalent symptoms affected by Long-COVID and concluded that: “While this study does not provide recommendations for specific therapies, in the absence of approved treatments, insights from patient-reported experiences provide urgently needed real-world evidence for developing targeted patient care therapies and future clinical trials” (Figure 5) [94].

### 5.5. Future Research Directions

Future studies should integrate multi-omics approaches—combining transcriptomics, proteomics, and metabolomics—with advanced imaging and minimally invasive muscle biopsies (TPNBs) to characterize disease progression at the molecular level. Video imaging techniques have been tested in an attempt to characterise the severity of ME/CFS, allowing for stratification to guide the path to recovery. To achieve this aim, Almutairi et al. conducted a systematic review of published studies using imaging techniques as MRI in ME/CFS patients, but did not reach any definitive conclusions [71]. Very recently, the development of new sensors used in near-infrared spectroscopy (NIRS), a technique that allows comparison with data obtained with the aid of NMR, leaves the door open to results that are more useful for a definite diagnosis, at least of the state of fatigue typical of the disease [72].

Another useful example of video imaging techniques being used to study stratification—which is necessary for defining the severity of ME/CFS—concerns the hypothesis that neuroinflammation is present in specific areas of the CNS, and is causally linked to the severity experienced by ME/CFS patients [95].

This hypothesis has been supported by the demonstration of a state of cerebral neuroinflammation in CFS/ME patients using (11)C-(R)-PK11195 and PET. This technique demonstrated that the binding potential (BP(ND)) was 45% to 200% higher in the cingulate cortex, hippocampus, amygdala, thalamus, midbrain and pons of CFS/ME patients than in healthy controls [74].

Longitudinal monitoring of muscle function in ME/CFS and Long COVID cohorts could elucidate the transition from post-viral fatigue to chronic metabolic myopathy. Furthermore, exploring the H_2_O_2_/Ca^2+^/Zn^2+^ signaling triad as a redox-sensitive regulatory hub may offer insights into mitochondrial adaptability and its failure in ME/CFS. Ultimately, the goal is to move beyond symptomatic descriptions toward a mechanistically informed nosology, where ME/CFS is recognized as a spectrum disorder arising from a continuum of mitochondrial, redox, and calcium-related dysfunctions.

In conclusion, although we claim that skeletal muscle alterations play a significant role in the pathophysiological changes typical of ME/CFS, this does not mean that all consequences of this functional state in affected individuals should be attributed to these alterations. ME/CFS remains a multifactorial disease with multiple aetiopathogenetic and structural factors. However, in this context, it is necessary to reassess the role of skeletal muscle. This is because it is both an endocrine producer of signals and a mitochondrial generator of energy and accumulator of oxidative stress.

## Figures and Tables

**Table 1:** Comparative Analysis of Diagnostic Criteria: CDC vs. CCC vs. IOM/SEID.

Feature	CDC (1994)	CCC (2003)	IOM/SEID (2015)
Primary Focus	Unexplained chronic fatigue.	Neurological and Immunological features.	Post-Exertional Malaise (PEM) and core symptoms.
Pathognomonic Sign (PEM)	Optional (one of 8 minor criteria).	Mandatory.	Mandatory.
Sensitivity	High. Identifies a broad and heterogeneous patient population.	Moderate. More restrictive; excludes patients without systemic involvement.	High. Designed for clinical utility and inclusivity of severe cases.
Specificity	Low. High risk of misclassifying primary psychiatric or other fatiguing conditions.	High. Highly effective at distinguishing ME from other pathologies.	Moderate/High. More specific than Fukuda, though less granular than CCC.
Application	Former gold standard.	Clinical research and rigorous diagnosis.	Rapid clinical diagnosis (Primary Care).

**Table 2:** Pathophysiological alterations of the skeletal muscle secretome in ME/CFS.

Secretome Component	Pathological Deviation	Pathophysiological Mechanism	Clinical Aspects
Pro-inflammatory Myokines	Post-exertional elevation/Prolonged expression	Systemic Inflammation: Activation of the HPA axis and systemic immune response.	Flu-like symptoms and systemic PEM.
Extracellular Vesicles (EVs)	Altered cargo (miRNAs, mtDNA)	Intercellular Communication: Transport of “danger signals” to the Central Nervous System.	Neuroinflammation and cognitive impairment (“brain fog”).
Lactate & Metabolic Acids	Accelerated accumulation/Impaired clearance	Peripheral Sensitization: Irritation of muscle nociceptors (acid-sensing ion channels).	Muscle pain and deep somatic hyperalgesia.
Reactive Oxygen Species (ROS)	Excessive production vs. low antioxidant capacity	Oxidative Stress: Damage to mitochondrial membranes and proteins.	Non-restorative fatigue and cellular energy depletion.
Growth Factors (e.g., IGF-1, BDNF)	Reduced bioavailability or signaling	Impaired Recovery: Decreased regenerative capacity and neuroplasticity.	Prolonged recovery time (lasting days or weeks).

The table synthesizes the dysregulation of muscle-derived signaling molecules (myokines, metabolites, and EVs following exertional triggers). In ME/CFS, the skeletal muscle secretome shifts from a homeostatic regenerative profile to a pro-inflammatory and oxidative state. This biochemical shift facilitates the transition from peripheral exertion to systemic symptomatic exacerbation. Abbreviations: BDNF, brain-derived neurotrophic factor; HPA, hypothalamic–pituitary–adrenal; IGF-1, insulin-like growth factor 1; mtDNA, mitochondrial DNA; miRNA, microRNA.

## Data Availability

No new data were created or analyzed in this study. Data sharing is not applicable to this article.
